# Clinical Characteristics and Hypokalemia Risk Prediction in Patients With Hyperosmolar Hyperglycemic State and Diabetic Ketoacidosis (HHS-DKA): A Single-Center, Retrospective Observational Study

**DOI:** 10.7759/cureus.84672

**Published:** 2025-05-23

**Authors:** Yuichiro Iwamoto, Tomohiko Kimura, Yuichi Morimoto, Ayaka Harada, Kazunori Dan, Hideyuki Iwamoto, Yoshiro Fushimi, Junpei Sanada, Masashi Shimoda, Shuhei Nakanishi, Tomoatsu Mune, Kohei Kaku, HIdeaki Kaneto

**Affiliations:** 1 Department of Diabetes, Endocrinology and Metabolism, Kawasaki Medical School, Kurashiki, JPN; 2 Department of Pediatrics, Kindai University, Osakasayama, JPN

**Keywords:** continuous insulin infusion therapy, diabetes mellitus, diabetic ketoacidosis (dka), hyperosmolar hyperglycemic state (hhs), retrospective observational study

## Abstract

Background: People with combined diabetic ketoacidosis (DKA) and hyperosmolar hyperglycemic state (HHS) often present with more severe metabolic derangements than those with DKA or HHS alone. This study aimed to clarify the clinical characteristics of HHS-DKA and explore predictive models for complications, including hypokalemia.

Methods: We retrospectively analyzed data from 99 patients admitted with hyperglycemic emergencies between April 1, 2010, and October 31, 2024, and classified them into DKA, HHS, and HHS-DKA groups. A decision tree model was also developed to predict the risk of post-continuous insulin infusion (CII) hypokalemia. The decision tree model was created using machine learning with the Python language (Python Software Foundation, Wilmington, Delaware).

Results: HHS-DKA patients had significantly higher rates of acute kidney injury (84%) and hyperkalemia (58%) compared to those with DKA or HHS alone. A decision tree model predicted post-CII hypokalemia with 80% accuracy, identifying key predictors such as initial blood glucose and insulin flow rates.

Conclusion: HHS-DKA represents a distinct and severe clinical entity with unique characteristics and complications. Predictive models developed in this study will likely assist in risk stratification and improve patient management during hyperglycemic crises in emergency settings. However, as this was a single-center retrospective study without external validation, further studies are warranted to confirm these findings.

## Introduction

Hyperosmolar hyperglycemic state (HHS) and diabetic ketoacidosis (DKA) are both serious metabolic complications associated with diabetes and can be encountered in emergency care settings [1]. HHS, typically seen in older patients with type 2 diabetes, is characterized by severe dehydration caused by hyperglycemia and osmotic diuresis [2,3]. In contrast, DKA is more common in individuals with type 1 diabetes and is caused by absolute or relative insulin deficiency, leading to ketoacidosis due to the accumulation of ketone bodies [4,5].

While these conditions have distinct pathophysiologies, they may overlap in clinical practice. Cases involving features of both, commonly referred to as combined HHS and DKA (HHS-DKA), have been associated with increased morbidity and mortality [6]. Approximately 27% of patients presenting with DKA may also meet criteria for HHS [7], and the diagnostic distinction can be difficult in real-world settings due to overlapping clinical and laboratory findings. Despite their clinical relevance, few studies have systematically described the characteristics and treatment responses of patients with HHS-DKA, particularly in Asian populations.

As hyperglycemic crises become increasingly common, especially among older patients and those receiving SGLT2 inhibitors, understanding the unique features and management challenges of HHS-DKA is essential. This retrospective study aimed to describe the clinical characteristics and complications of patients with HHS-DKA compared to those with DKA or HHS alone, and to explore predictors of hypokalemia during treatment using machine learning methods.

The results of this study were presented in an abstract at the 68th Annual Meeting of the Japan Diabetes Society in Okayama.

## Materials and methods

Study subjects

Kawasaki Medical School Hospital is a tertiary emergency care center staffed by board-certified diabetologists who can manage hyperglycemic emergencies. The subjects of this study were 103 patients with diabetes mellitus who were hospitalized in the Department of Diabetes, Endocrinology, and Metabolism at Kawasaki Medical School Hospital and received continuous insulin infusion (CII) between April 1, 2010, and October 31, 2024. Among these patients, four patients with normal osmolarity and no ketoacidosis were excluded from the analysis. Ultimately, 99 patients who received CII were included in the analysis. In this study, cases of diabetes with hyperglycemia complicated by ketosis and metabolic acidosis were diagnosed as DKA (n=14). Cases of diabetes with hyperglycemia complicated by a hyperosmolar state were diagnosed as HHS (n=35). Cases of diabetes with hyperglycemia complicated by a hyperosmolar state and ketoacidosis were diagnosed as HHS-DKA (n=50).

Study designs

This study is a single-center, retrospective cohort study. All data for analysis were collected from medical records. Physical measurements recorded age, height, weight, body mass index (BMI), and vital signs during hospitalization. Comorbidities, duration of diabetes, smoking history, and drinking history were obtained by interview. The main complaint was also recorded at the time of admission, and for participants with multiple main complaints, each was treated as a binary variable. The blood tests described in this study were performed before CII was started. All subjects underwent CII after hospitalization. The insulin flow rate, type, and dose of infusion fluid were determined at the attending physician's discretion.

Definition of each disease and its complications

The diagnostic criteria for diabetes mellitus were a past diagnosis of diabetes mellitus or, in the absence of a past diagnosis of diabetes mellitus, a blood glucose level of 200 mg/dL or higher at any time, plus a glycosylated hemoglobin (HbA1c) level of 6.5% or higher or symptoms related to hyperglycemia. This study's diagnostic criteria for ketosis were defined as a blood total ketone level of 200 μmol/L or higher, the institutional standard, and acidosis was described as an arterial blood pH of less than 7.35. Hyperosmolarity was defined as a plasma osmolality of 320 mOsmol/kg or higher, calculated with blood glucose using corrected sodium, where corrected sodium was calculated by (serum sodium (mmol/L)) + ((blood glucose (mg/dL) - 100) * 1.6/100) [1]. The osmolality was calculated by (corrected sodium (mmol/L)) * 2 + (blood glucose (mg/dL))/18 + (urea nitrogen (mg/dL))/2.8. The diagnostic criteria for DKA were patients with diabetes mellitus who had combined ketosis and acidosis at the start of treatment; the diagnostic criteria for HHS were patients with diabetes mellitus complicated by hyperosmolarity and without acidosis [5]; the diagnostic criteria for HHS-DKA were patients with diabetes mellitus complicated by hyperosmolarity and ketoacidosis.

The definition of complications is given below. If a participant had multiple complications of diabetes, each was counted as one complication. Acute kidney injury (AKI) was defined according to the KDIGO (Kidney Disease: Improving Global Outcomes) criteria [8], which include an increase in serum creatinine by ≥0.3 mg/dL within 48 hours or an increase of ≥1.5 times of baseline within seven days, or urine output <0.5 mL/kg/h for six hours. Hyperkalemia was defined as serum potassium ≥ 5.5 mmol/L. Diagnosis of gastroenteritis requires confirmation of clinical symptoms (diarrhea, vomiting, abdominal pain) and abnormal findings on endoscopy or computed tomography (CT). Acute necrotizing esophagitis was defined as black vomiting and findings of a black esophagus on gastrointestinal endoscopy. Rhabdomyolysis was defined as elevated levels of myogenic enzymes (CK > 1000 U/L), positive urinary occult blood reaction, and negative erythrocytes on sedimentation. Mediastinal emphysema and pancreatitis were diagnosed when there were associated symptoms and findings on CT. Metabolic encephalopathy was defined as the presence of findings on magnetic resonance imaging of the head and an abnormal electroencephalogram. Mortality was defined as the percentage of patients who died during the hospital stay. Hypokalemia was defined as mild hypokalemia if 3.0-3.5 mmol/L and severe hypokalemia if <3.0 mmol/L. Refeeding syndrome was diagnosed when a rapid drop in potassium, magnesium, and phosphorus occurred after carbohydrate-containing intravenous fluids or oral or enteral nutrition initiation. All cases diagnosed with gastrointestinal tract-related complications after treatment were diagnosed by gastrointestinal endoscopy.

Statistical analysis

Data are expressed as median (interquartile). This study aimed to characterize the clinical features of patients with combined HHS-DKA compared to those with DKA or HHS alone, and to develop a model for predicting hypokalemia during CII. The primary outcomes were the incidence and type of complications observed in each group (e.g., AKI, hypokalemia, and myocardial infarction). The secondary outcome was the identification of clinical predictors for hypokalemia using a decision tree-based machine learning model. To evaluate the differences in patient background among the three groups, the Steel-Dwass test and chi-square test were used. The Wilcoxon rank sum test was used to assess the differences in the presence or absence of complications during hospitalization, blood glucose levels, total ketone bodies, serum osmolality, and bicarbonate ion concentrations. A decision tree for predicting the risk of hypokalemia after CII initiation was created using machine learning. Clinical data obtained from medical records were used as training data for machine learning. The data used in this study did not contain any missing values. Of the clinical data obtained from the study participants, 70% (n=69) were randomly assigned to the training data and 30% (n=30) to the test data. The decision tree model was trained using a maximum depth of X and Gini impurity as the splitting criterion. Five-fold cross-validation was used to prevent overfitting. Receiver operating characteristic (ROC) and area under the curve (AUC) assessed model performance. The statistical significance level in this study was set at p < 0.05. JMP Pro (17.0.0, JMP Statistical Discovery LLC, Cary, North Carolina) was used for the analysis. The graphs were created by Excel (16.92) for Mac (Microsoft Corporation, Redmond, Washington) and Python version 3.10.12 (Python Software Foundation, Wilmington, Delaware). Python version 3.10.12 was used as the programming language, and scikit-learn version 1.6.0 was used for clinical model creation.

Ethical considerations

The research protocol, including the opt-out system for informed consent, was approved by the Ethics Committee of Kawasaki Medical School (No. 6229-00). The Declaration of Helsinki was followed in conducting this study. As this was a retrospective study, instead of obtaining informed consent from each patient, information about the study was disclosed via the hospital website of each facility. This study was conducted under the approved IRB protocol titled “Development of Predictive Models for the Treatment of Hyperglycemic Emergencies Using Artificial Intelligence,” which includes DKA, HHS, and soft drink ketosis as target conditions. The approved protocol explicitly covers retrospective clinical data to analyze treatment response and build predictive models (including machine learning components).

## Results

Clinical characteristics

The clinical characteristics of the subjects in this study are shown in Table 1. There were no differences in age, gender, duration of diabetes, or length of hospital stay among the three groups. The proportion of cases in which insulin therapy was administered at the time of hospitalization was significantly higher in DKA (35.7%) and HHS-DKA (34.0%) than in HHS (9.1%) (p=0.018). The rate of insulin therapy at the time of discharge was 71.4% for DKA, 60.6% for HHS, and 66.0% for HHS-DKA, with no difference among the three groups. Regarding medical history, previous malignant tumors were significantly higher in the HHS group (p=0.028). The three groups did not differ in the prevalence of other diabetes-related complications. The chief complaints at the time of admission are shown in Table 2. The proportion of patients with impaired consciousness as the chief complaint was higher in the HHS-DKA group (48.0%) than in the DKA (14.3%) and HHS (28.6%) groups (p=0.034). The proportion of patients whose main complaint was a gastrointestinal symptom, such as nausea, vomiting, or abdominal pain, tended to be higher in DKA (50.0%) than in HHS (17.1%) or HHS-DKA (24.0%) (p=0.055).

**Table 1:** Clinical characteristics of the subjects in this study. Data presented as median (interquartile range). Kruskal-Wallis test and chi-square test were used for analysis. The Steel-Dwass test was used as the between-group test. * p < 0.05 (vs. DKA). DKA, diabetic ketoacidosis; HHS, hyperosmolar hyperglycemic state; SDR, mild non-proliferative diabetic retinopathy; PPDR, pre-proliferative retinopathy; PDR, proliferative diabetic retinopathy.

Parameters	DKA (n=14)	HHS (n=35)	HHS-DKA (n=50)	p-value
Male/female	7/7	25/10	26/24	0.157
Age (years)	41 (31-58)	66 (52-77)	62 (44-73)	0.204
Duration of diabetes (years)	6 (2-19)	2 (0-10)	9 (0-18)	0.191
Hospitalization period (days)	15 (9-16)	16 (14-21)	17 (13-24)	0.144
Insulin therapy on admission (n (%))	5 (35.7)	3 (9.1)*	17 (34.0)	0.018
Insulin therapy at the time of discharge (n (%))	10 (71.4)	21 (60.6)	33 (66.0)	0.645
SGLT2 inhibitor usage on admission (n (%))	1 (7.1)	2 (5.7)	6 (12.0)	0.589
Oral steroid medication on admission (n (%))	0 (0)	4 (11.4)	3 (6.0)	0.339
Antipsychotic medication on admission (n (%))	2 (14.3)	2 (5.7)	3 (6.0)	0.524
Smoking history (past/current, n)	0/4	8/10	5/15	0.218
Drinking history (ethanol <20 g/day/>20 g/day, n)	3/1	5/4	13/8	0.555
History of ischemic heart disease (n (%))	0 (0)	4 (11.4)	2 (4.0)	0.218
History of stroke (n (%))	1 (7.1)	4 (11.4)	1 (2.0)	0.197
History of cancer (n (%))	0 (0)	9 (25.7)	7 (14.0)	0.028
History of diabetic neuropathy (n (%))	7 (50.0)	11 (31.4)	39 (43.8)	0.688
Diabetic retinopathy (SDR/PPDR/PDR, n)	1/1/3	0/0/2	4/0/2	0.053
Diabetic nephropathy (Stage 1/2/3/4, n)	7/5/2/0	16 /13/4/2	23/16/3/7	0.625
Height (cm)	163 (156-170)	162 (155-168)	160 (150-171)	0.851
Body weight (kg)	61 (48-74)	56 (46-72)	54 (43-66)	0.363
Body mass index (kg/m^2^)	23.0 (17.5-27.7)	22.4 (19.4-26.4)	21.7 (17.9-24.9)	0.370
Systolic blood pressure (mmHg)	135 (106-149)	134 (118-145)	134 (110-155)	0.993
Diastolic blood pressure (mmHg)	86 (68-96)	82 (76-93)	77 (62-85)	0.064
Pulse rate (beats per minute)	106 (97-120)	98 (82-111)	108 (90-121)	0.110
Body temperature (℃)	36.9 (36.5-37.3)	36.5 (36.4-37.2)	36.5 (36.2-37.1)	0.325

**Table 2:** Chief complaints and complications on admission of the subjects in this study. Chi-square tests were used for analysis. Symptoms and triggers were recorded separately, and overlaps were allowed. DKA, diabetic ketoacidosis; HHS, hyperosmolar hyperglycemic state; COVID-19, coronavirus disease 2019.

Chief complaints or complications	DKA (n=14)	HHS (n=35)	HHS-DKA (n=50)	p value
Disturbance of consciousness (n (%))	2 (14.3)	10 (28.6)	24 (48.0)	0.034
Gastrointestinal symptoms (n (%))	7 (50.0)	6 (17.1)	12 (24.0)	0.055
Hyperglycemic symptoms (n (%)	6 (42.9)	18 (51.4)	16 (32.0)	0.195
Weight change (increased/decreased, n)	0/6	1/17	1/23	0.593
Heavy soft drink consumption (n (%))	5 (35.7)	22 (62.9)	28 (56)	0.224
Shock vitals on admission (n (%))	0 (0)	1 (2.9)	2 (4.0)	0.740
Triggers	Soft drink (n=5), infection (n=5), anorexia nervosa (n=1), unknown (n=3)	Soft drink (n=18), infection (n=9), unknown (n=8)	Soft drink (n=18), infection (n=20), anorexia nervosa (n=1), suspected adverse reaction of COVID-19 vaccine (n=1), interruption of insulin therapy (n=1), unknown (n=8)	-
Prevalence of any complications (n (%))	9 (64.3)	25 (71.4)	48 (96.0)	0.002
Infection (n (%))	5 (35.7)	9 (25.7)	20 (40.0)	0.391
Acute kidney injury (n (%))	4 (28.6)	20 (57.1)	42 (84.0)	<0.001
Hyperkalemia (n (%))	4 (28.6)	11 (31.4)	29 (58.0)	0.023
Gastroenteritis (n (%))	7 (50.0)	6 (17.1)	12 (24.0)	0.072
Acute necrotizing esophagitis (n (%))	1 (7.1)	2 (5.7)	8 (16.0)	0.292
New onset macrovascular obstruction (n (%))	0 (0)	1 (2.9)	4 (8.0)	0.367
New onset atrial fibrillation (n (%))	0 (0)	2 (5.7)	2 (4.0)	0.656
Rhabdomyolysis (n (%))	0 (0)	1 (2.9)	2 (4.0)	0.740
Mediastinal emphysema (n (%))	0 (0)	0 (0)	3 (6.0)	0.220
Pancreatitis (n (%))	0 (0)	0 (0)	2 (4.0)	0.368
Metabolic encephalopathy (n (%))	0 (0)	1 (2.9)	3 (6.0)	0.546

Causes of hyperglycemic emergency and complications on admission

The causes of hyperglycemic emergency are shown in Table 2. Excessive consumption of soft drinks or infectious diseases were common triggers of hyperglycemic emergencies in the three groups. The causes of DKA infections were methicillin-sensitive *Staphylococcus aureus* (MSSA) sepsis (n=1), pneumonia of unknown origin (n=1), influenza virus infection (n=2), and COVID-19 infection (n=1). The causes of infection in the HHS were sepsis (*Escherichia coli*, *Proteus mirabilis*, and MSSA, n=1 each), urinary tract infection (*E. coli*, *Enterococcus faecalis*, and *Klebsiella pneumoniae*, n=1 each), aspiration pneumonia (n=2), and upper respiratory tract inflammation (n=1). The causes of infection in HHS-DKA were sepsis (MSSA (n=3), methicillin-resistant *Staphylococcus aureus* (MRSA), *E-coli*, and unknown causative bacteria, n=1 each), urinary tract infection (*Candida*, MSSA, and unknown causative bacteria, n=1 each), pneumococcal pneumonia (n=2), COVID-19 infection (n=2), influenza (n=1), invasive pulmonary aspergillosis (n=1), aspiration pneumonia (n=2), acute prostatitis (n=1), infection with pathogenic *E. coli* O125 (n=1), and bacterial enteritis (n=1).

Acute complications at the time of hospitalization were more common in HHS (71.4%) than in DKA (64.3%), and the most common was in patients with HHS-DKA (96.0%). Among the three groups, the HHS-DKA group showed the highest frequency of AKI and hyperkalemia (p<0.001 and p=0.023, respectively), indicating a significantly higher severity of hyperglycemic emergencies in these cases. Gastroenteritis tended to occur more frequently in the DKA group (p=0.072). However, there was no statistical difference in other complications among the three groups; more severe complications were observed in the HHS and HHS-DKA groups compared to the DKA group.

Blood tests on admission

The results of blood tests on admission of the subjects in this study are shown in Table 3. The number of white blood cells at hospitalization was significantly higher in the HHS-DKA group than in the DKA or HHS alone group (p=0.007). In order, renal function decreased in the DKA, HHS, and HHS-DKA groups. Blood glucose levels were significantly higher in the DKA group (377 (328-551) mg/dL) than in the HHS group (708 (537-926)) and the HHS-DKA group (737 (599-915)) (p<0.001). There was no difference in HbA1c levels among the three groups. Total ketone bodies were significantly higher in DKA and HHS-DKA groups than in the HHS group. At the same time, pH, bicarbonate ion concentration, and base excess were substantially lower in DKA and HHS-DKA groups than in the HHS group. On the other hand, 81.8% of HHS cases showed ketosis without acidosis.

**Table 3:** Blood tests on admission of the subjects in this study. Data presented as median (interquartile range). Kruskal-Wallis test and chi-square test were used for analysis. The Steel-Dwass test was used as the between-group test. * p < 0.05 (vs. DKA); ♯ p < 0.05 (HHS vs. HHS-DKA). DKA, diabetic ketoacidosis; HHS, hyperosmolar hyperglycemic state; AST, aspartate aminotransferase; ALT, alanine aminotransferase; eGFR, estimated glomerular filtration rate; NGSP, National Glycohemoglobin Standardization Program.

Parameters	DKA (n=14)	HHS (n=35)	HHS-DKA (n=50)	p-value
White blood cell (/μL)	10630 (9095-12535)	10600 (7365-12260)	13890 (8793-17645)^♯^	0.007
Red blood cell (x10^4^/μL)	504 (463-569)	461 (421-533)	466 (406-525)	0.191
Hemoglobin (g/L)	15.5 (13.9-16.7)	14.1 (12.6-15.8)	14.3 (12.5-16.0)	0.133
Hematocrit (%)	45.8 (40.7-50.6)	41.9 (38.5-45.8)	43.4 (38.6-49.6)	0.312
Platelet cell (x10^4^/μL)	27.5 (20.7-34.7)	21.0 (17.0-25.4)	23.6 (19.0-31.6)	0.125
Albumin (mg/dL)	4.4 (3.7-4.6)	3.8 (3.2-4.2)	3.9 (3.5-4.3)	0.126
AST (IU/L)	19 (16-33)	22 (14-41)	23 (14-32)	0.753
ALT (IU/L)	25 (18-63)	19 (14-49)	22 (16-31)	0.532
Urea nitrogen (mg/dL)	17 (12-36)	34 (15-45)	42 (29-71)*^♯^	<0.001
eGFR (mL/min/1.73m^2^)	87 (58-105)	51 (36-79)*	39 (20-53)*^♯^	<0.001
Creatinine (mg/dL)	0.8 (0.5-1.1)	1.1 (0.7-1.5)*	1.3 (1.0-2.4)*^♯^	<0.001
Uric acid (mg/dL)	8.8 (6.6-10.4)	6.4 (5.7-10.7)	10.4 (8.1-15.4)^♯^	<0.001
C-reactive protein (mg/dL)	0.83 (0.23-2.08)	0.99 (0.20-2.46)	1.24 (0.47-4.91)	0.294
Blood glucose (mg/dL)	377 (328-551)	708 (537-926)*	737 (599-915)*	<0.001
Hemoglobin A1c (NGSP, %)	12.7 (10.2-13.5)	12.1 (9.6-14.7)	10.9 (9.5-12.3)	0.130
Total ketone body (μmol/L)	6920 (2421-10863)	1523 (793-5161)	12590 (5055-15380)^♯^	<0.001
Acetoacetic acid (μmol/L)	2199 (813-2878)	494 (243-1632)*	3365 (1930-4690)^♯^	<0.001
3-hydroxybutyric acid (μmol/L)	4648 (1665-6344)	1009 (488-3529)	8680 (3853-11235)^♯^	<0.001
Sodium (mmol/L)	126 (121-136)	135 (128-140)	136 (131-141)	0.252
Potassium (mmol/L)	4.6 (4.3-5.0)	4.6 (4.0-5.2)	5.2 (4.3-5.8)^♯^	0.045
Chlorine (mmol/L)	97 (90-100)	95 (88-103)	97 (89-105)	0.776
Serum osmolality (mOsm/kg)	295 (288-299)	330 (303-347)*	341 (319-360)^♯^	<0.001
pH	7.28 (7.13-7.30)	7.39 (7.37-7.42)*	7.24 (7.09-7.29)^♯^	<0.001
Bicarbonate ion concentration	11 (5-19)	22 (18-24)*	9 (5-14)^♯^	<0.001
Base excess (mEq/L)	-13 (-22 to -6)	-2 (-6 to 0)*	-16 (-23 to -11)^♯^	<0.001

Reactivity to CII and complications that occurred after the start of treatment

**Figure 1:**
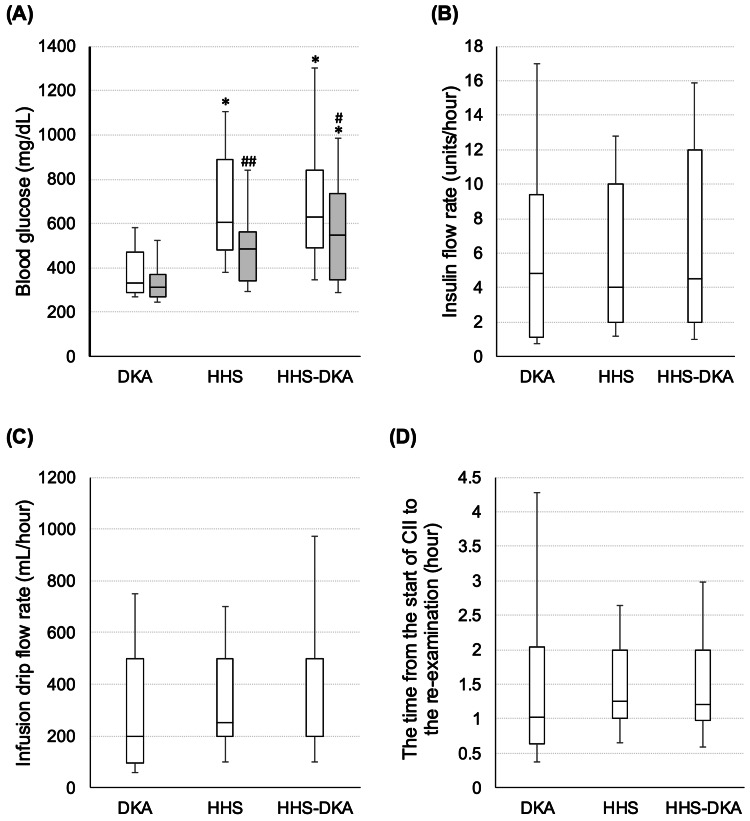
Reactivity after CII initiation in the three groups: DKA alone, HHS alone, and DKA-HSS combination. (A) Blood glucose level before CII (white box) and re-checked blood glucose level after CII (gray box). (B) Insulin flow rate from the start of CII to a re-examination of blood glucose level. (C) Infusion drip flow rate from the start of CII to the time of re-examination of blood glucose level. (D) Time from the beginning of CII to the time of re-examination of blood glucose level. * p < 0.05 vs. DKA, # p < 0.05; ## p < 0.005 before vs. after CII. The Kruskal-Wallis test was used for analysis, and the Steel-Dwass test was used as a post-hoc test. CII, continuous insulin infusion; DKA, diabetic ketoacidosis; HHS, hyperosmolar hyperglycemic state.

Before CII, 11 patients with DKA, 18 with HHS, and 31 with HHS-DKA received intravenous saline or Ringer's acetate. Before CII, blood glucose levels were 330 (290-473) mg/dL in DKA, 605 (481-888) mg/dL in HHS, and 629 (491-842) mg/dL in the DKA-HHS group, respectively (Figure 1A). There was no difference in the insulin or infusion drip flow rate at the start of CII among the three groups (p=0.689 and p=0.099, respectively; Figures 1B, 1C). The time from the beginning of CII to the re-examination of blood glucose levels was 1.0 (0.6-2.0) hour for DKA, 1.3 (1.0-2.0) hours for HHS, and 1.2 (1.0-2.0) hours for HHS-DKA, with no difference among the three groups (p=0.750; Figure 1D). The re-examined blood glucose level was significantly higher in the HHS-DKA group (547 (345-735) mg/dL) than in the DKA (315 (270-373) mg/dL) and HHS (485 (340-561) mg/dL) groups (p<0.001; Figure 1A). In the HHS and HHS-DKA groups, blood glucose levels were significantly reduced after the start of CII (p=0.0018 and p=0.048, respectively). The difference between the initial blood glucose level and the re-examined blood glucose level was -44 (-108-23) for the DKA group and -77 (-203 to -16) for the HHS-DKA group, with no difference (p=0.235), but -159 (-219 to -66) for the HHS group, which was significantly lower than the DKA group (p=0.005).

Table 4 shows the complications that occurred after the start of CII. The proportion of patients who experienced some complication was 35.7% for DKA, 31.4% for HHS, and 40.0% for the HHS-DKA group, with no difference among the three groups. The proportion of patients who died during hospitalization was 0% for DKA, 2.9% for HHS, and 8.0% for HHS-DKA. Hypokalemia occurred in 31.3% of participants and was the most frequent post-treatment complication, but there was no difference in the incidence of this complication among the three groups.

**Table 4:** Complications that arose after the start of CII. Chi-square tests were used for analysis. CII, continuous insulin infusion; DKA, diabetic ketoacidosis; HHS, hyperosmolar hyperglycemic state.

Complications	DKA (n=14)	HHS (n=35)	HHS-DKA (n=50)	p-value
Prevalence of complications (n (%))	5 (35.7)	11 (31.4)	20 (40.0)	0.720
Mortality rate (n (%))	0 (0)	1 (2.9)	4 (8.0)	0.367
Hypokalemia (3.0-3.5 mmol/L/<3.0 mmol, n)	3 (21.4)/2 (14.3)	8 (22.9)/1 (2.9)	13 (26.0)/4 (8.0)	0.670
Refeeding syndrome (n (%))	0 (0)	1 (2.9)	2 (4.0)	-
Pseudomembranous enteritis (n (%))	0 (0)	1 (2.9)	1 (2.0)	-
Ileal necrosis (n (%))	0 (0)	1 (2.9)	0 (0)	-
Duodenal ulcer (n (%))	0 (0)	0 (0)	1 (2.0)	-
Post-resuscitation encephalopathy (n (%))	0 (0)	0 (0)	1 (2.0)	-
Heparin-induced thrombocytopenia (n (%))	0 (0)	0 (0)	1 (2.0)	-

Factors associated with complications on admission

**Figure 2:**
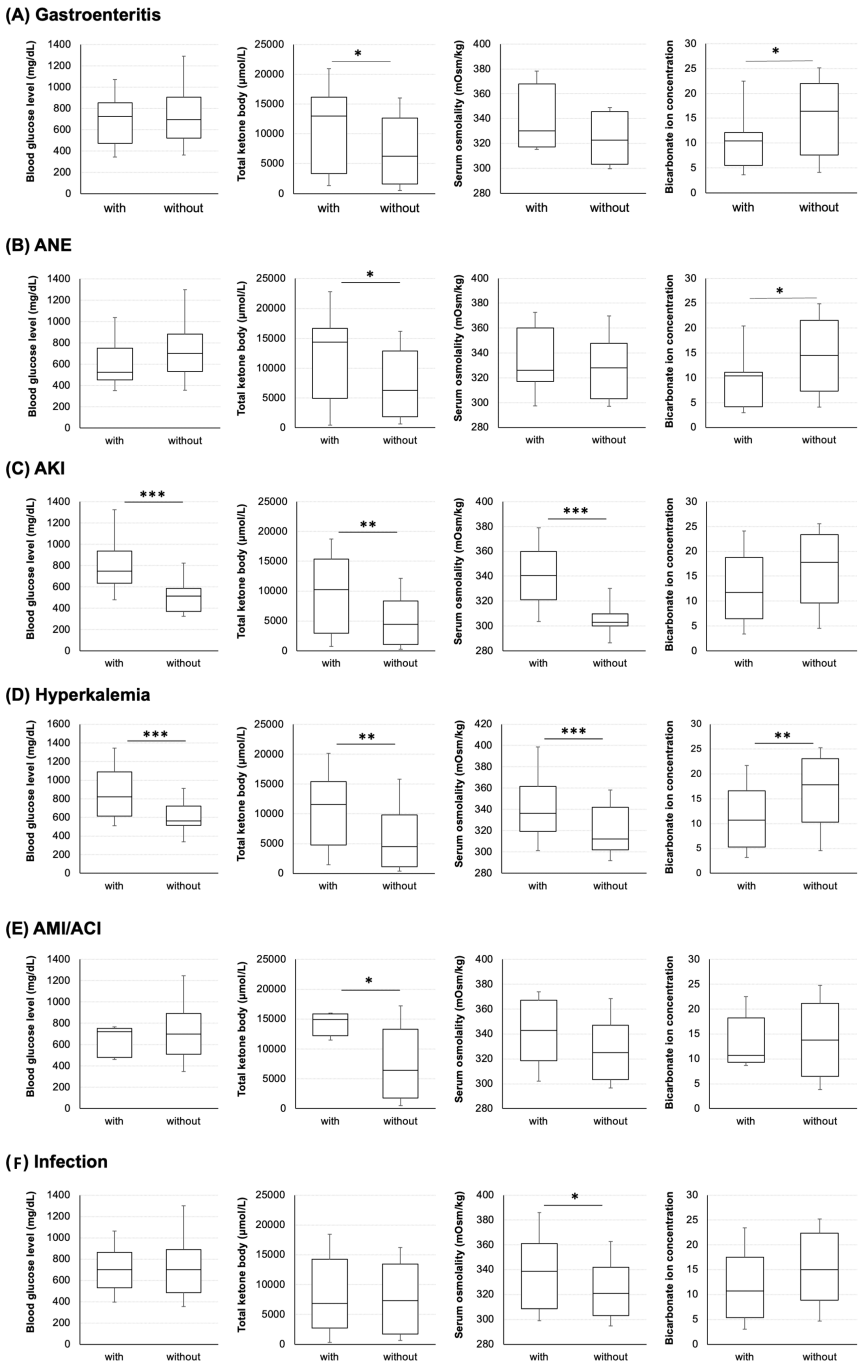
Correlation with various complications and clinical parameters. On admission, various complications were associated with blood glucose, total ketones, serum osmolality, and bicarbonate ion concentration. (A) Gastroenteritis, (B) acute necrotizing esophagitis (ANE), (C) acute kidney injury (AKI), (D) hyperkalemia, (E) acute myocardial infarction (AMI) or acute cerebral infarction (ACI), and (F) infection. * p = 0.05, ** p = 0.005, and *** p = 0.001. The Wilcoxon signed-rank test was used for analysis.

The results of the evaluation of factors related to acute and post-treatment complications are shown in Figure 2. The total ketone body levels in cases with concomitant gastroenteritis at admission were 12,965 (3,323-16,163) μmol/L, whereas those in cases without concomitant gastroenteritis were 6,257 (1,604-12,670) μmol/L, which was significantly higher. Additionally, the bicarbonate ion concentration was 16.4 (7.6-22.0) mmol/L in cases without gastroenteritis and 10.4 (5.5-12.1) mmol/L in cases with gastroenteritis, which was significantly lower (p=0.436 and p=0.229, respectively; Figure 2A). This trend was also observed in acute necrotizing esophagitis (ANE) (Figure 2B). In addition, 28.7% of participants without gastroenteritis had a history of alcohol consumption, compared to 57.9% of participants with gastroenteritis. On the other hand, there was no difference in serum osmolality between those with and without gastroenteritis (p=0.156). Total ketone bodies in cases presenting with AKI or hyperkalemia at admission were 10,210 (2,940-15,340) μmol/L and 11,570 (4,761-15,400) μmol/L, respectively, significantly higher than in cases without these conditions (4,474 (1,075-8,352) and 4,474 (1,131-9,830) μmol/L; Figure 2C). Plasma osmolarity was also significantly higher in cases with AKI or hyperkalemia than those without these conditions (p<0.001; Figure 2D). Additionally, the bicarbonate ion concentration in cases with hyperkalemia was 107 (5.3-16.7) mmol/L, whereas it was 17.8 (10.3-23.1) mmol/L in non-complicated cases, indicating a significantly lower level in cases with hyperkalemia (p<0.001; Figure 2D). In cases with acute myocardial infarction or cerebral infarction, total ketone body was significantly higher (p=0.041; Figure 2E). In cases with infection, serum osmolality was considerably higher (p=0.041; Figure 2F). All of the deaths in this study were of male patients. Due to the low frequency of other complications, including deaths, no correlative factors could be identified in this study. To assess the potential influence of SGLT2 inhibitor use, we conducted a supplementary analysis excluding patients treated with SGLT2 inhibitors. The exclusion did not substantially alter the trends observed in key clinical outcomes such as AKI and mortality, supporting the robustness of the primary findings.

Factors associated with hypokalemia occurring after CII

**Figure 3:**
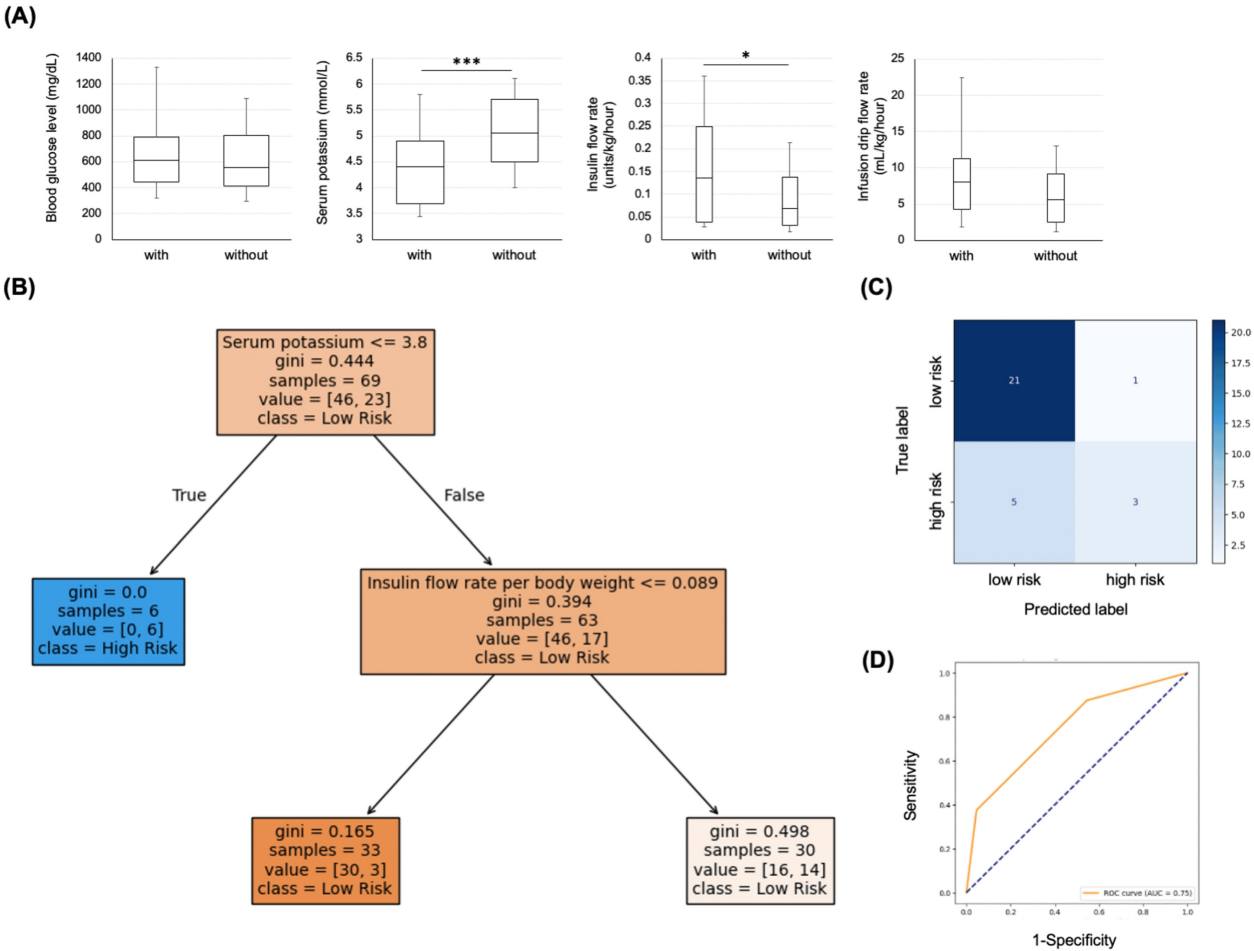
The decision tree was created to predict the risk of hypokalemia after CII initiation. (A) Correlation of hypokalemia after CII initiation with blood glucose, serum potassium, insulin flow rate per body weight, and infusion drip flow rate per body weight at CII initiation. * p < 0.05, *** p < 0.001. The Wilcoxon signed-rank test was used for analysis. (B) Decision tree for hypokalemia prediction after CII initiation. Each node shows the number of patients and the proportion of hypokalemia cases. (C) The confusion matrix shows the model’s performance in the test set, including sensitivity and specificity for high- and low-risk classifications. (D) The receiver operating characteristic (ROC) curve for the created decision tree model illustrates its performance across different thresholds. The decision tree was made using Python 3.10.12 and Scikit-learn version 1.6.0. CII, continuous insulin infusion.

In instances where hypokalemia occurred after CII was started, the serum potassium level at the start of treatment was significantly lower (p=0.003), and the insulin flow rate per body weight was significantly higher (p=0.013; Figure 3A). The time to hypokalemia with serum potassium levels of 3.0-3.5 mmol/L and less than 3.0 mmol/L was 3.9 (2.5-10.2) hours and 4.9 (2.5-9.0) hours, respectively, from the start of treatment. Figures 3B, 3C illustrate the decision tree model used to predict the risk of hypokalemia after initiating CII. The model stratifies patients based on three key variables: initial serum potassium level, insulin flow rate per body weight, and CRP level. The root node first splits at a potassium level of 3.8 mmol/L, followed by additional branches determined by insulin flow rate and CRP. Figure 3A shows the structure of the decision tree, including the thresholds for each split and the proportion of hypokalemia cases at each terminal node. Figure 3D summarizes the performance metrics of the model in the test dataset. The overall accuracy was 80%. The recall was 0.95 for the low-risk and 0.38 for the high-risk groups. Among cases classified as low risk, 81% were correctly predicted. We then applied the model to all study participants to explore its clinical implications. Among the 22 patients expected to be high risk but who did not develop hypokalemia, all cases with serum potassium ≤ 3.8 mmol/L were ultimately confirmed to develop hypokalemia and were thus correctly classified. In correctly classified cases, the median infusion drip flow rate per body weight was 5.8 (2.5-9.2) mL/kg/h, and the insulin flow rate was 0.068 (0.032-0.138) units/kg/h. In contrast, misclassified cases showed significantly higher infusion and insulin rates: 8.3 (5.4-11.4) mL/kg/h and 0.178 (0.093-0.321) units/kg/h, respectively (p = 0.047 and p < 0.001). Patients with serum potassium ≤ 3.8 mmol/L were at high risk of hypokalemia. In contrast, those with potassium > 3.8 mmol/L and an insulin flow rate ≤ 0.089 units/kg/h were identified as low-risk. Misclassification tended to occur in borderline potassium levels or cases receiving higher insulin doses. This highlights the importance of careful electrolyte monitoring in patients with borderline baseline potassium levels.

The amount of insulin and intravenous fluids needed to correct blood glucose levels

**Figure 4:**
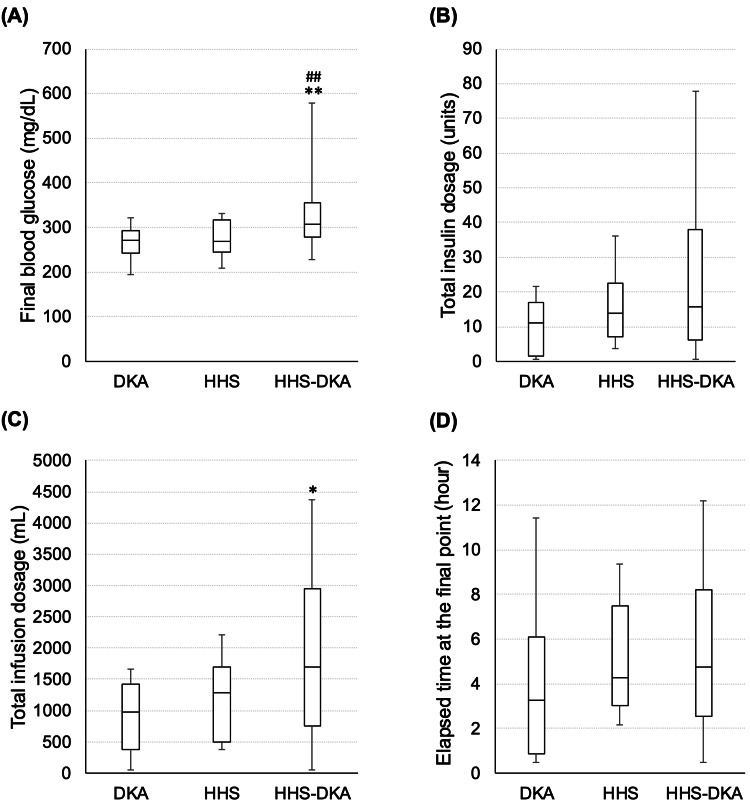
The final evaluation of CII in this study. The final evaluation was made when blood glucose levels were below 300 mg/dL or when oral intake was resumed, and the following were evaluated: (A) final blood glucose level, (B) total insulin dose, (C) total infusion dosage, and (D) time elapsed since the start of CII. * p < 0.05, ** p < 0.005 vs. DKA, # p < 0.05 vs. HHS. The Kruskal-Wallis test was used for analysis, and the Steel-Dwass test was used as a post-hoc test. CII, continuous insulin infusion; DKA, diabetic ketoacidosis; HHS, hyperosmolar hyperglycemic state.

Finally, the insulin and intravenous infusion doses required to reduce blood glucose levels to below 300 mg/dL or to the stage where oral intake can be resumed were evaluated. At the final evaluation, the blood glucose level was significantly higher in the HHS-DKA group than in the DKA and HHS groups (p=0.003; Figure 4A). There was no difference in the amount of insulin administered or the time elapsed from the start of treatment to the final evaluation between the three groups (p=0.18 and p=0.47, respectively; Figures 4B, 4D). On the other hand, the amount of intravenous fluid administered until the final evaluation was significantly higher in the HHS-DKA group than in the different groups (p=0.031; Figure 4C). The difference in blood glucose levels between the start of CII and the final evaluation was 60 (11-242) mg/dL in DKA, 327 (232-567) mg/dL in HHS, and 286 (143-515) mg/dL in HHS-DKA, showing that greater blood glucose reductions were seen in HHS and HHS-DKA compared to DKA (p<0.001 and p=0.005, respectively).

## Discussion

In this study, 51.5% of the patients in which CII was performed were cases of HHS-DKA. Compared to DKA or HHS alone, HHS-DKA was more frequently accompanied by renal dysfunction and other complications at admission, suggesting that it may be less responsive to treatment after the start of CII. Furthermore, we developed a clinical model to predict the risk of hypokalemia after the start of CII using machine learning based on the clinical data of the participants in this study. There have been few reports on the clinical characteristics of HHS-DKA or treatment guidelines using machine learning, and the results of this study are expected to help formulate treatment guidelines for emergency responses to hyperglycemic emergencies.

Known triggers of DKA include new onset of type 1 diabetes, insulin cessation, infection, and non-infectious causes such as acute myocardial infarction, alcohol, and pancreatitis [6,9]. Recurrent DKA is also known to be caused by insulin cessation, stress from chronic disease, and eating disorders [10]. HHS, on the other hand, is commonly caused by infections, acute cardiovascular disease, and other associated medical conditions [11,12]. Soft drink ketosis is ketoacidosis induced by consumption of sugary soft drinks and is often reported, especially in obese Asian men [13-15], but is rarely reported in other ethnic groups such as Caucasians [16]. While it has been thought that the mechanisms underlying the differences in incidence between ethnic groups are due to the difference in genetic background, this study was conducted in Japanese subjects, in which soft drinks were the most common triggers of hyperglycemic emergencies.

Although previously considered two distinct conditions, the overlap between DKA and HHS can substantially occur in clinical practice [17,18]. HHS-DKA had an adjusted odds ratio of 2.7 (95% CI: 1.4-4.9) for hospitalization and death compared to DKA and HHS alone, and an association of severe hypokalemia as a risk factor for death was reported [7]. Although there were few hospitalization deaths in this study and no statistically significant differences, hospitalization deaths were most common in HHS-DKA. In our study, 96.0% of DKA-HHS patients had some complications at the time of admission, with AKI (84%), hyperkalemia (58%), and infection (40%) being significantly more common than in the other groups. More frequent complications were observed in the DKA and HHS combination group compared to the DKA or HHS alone group (Table 2). These data suggest that the HHS-DKA combination is a more serious condition than DKA or HHS alone. To the best of our knowledge, this is the first report to compare the prevalence of each complication among patients with DKA alone, HHS alone, and DKA-HHS combination, while most reports on HHS-DKA have focused on in-hospital mortality.

The pathophysiology of HHS-DKA remains complex and may involve overlapping triggers and metabolic pathways. DKA and HHS are traditionally viewed as distinct entities, with DKA arising from absolute insulin deficiency and HHS from relative insulin deficiency coupled with profound dehydration. However, clinical observations and recent studies suggest that these two conditions may occur sequentially or concurrently, particularly in patients with delayed presentation, infection, or poor oral intake.

In some cases, DKA with severe dehydration may lead to hyperosmolality and fulfill the criteria for HHS, while in others, prolonged hyperglycemia and insulin resistance may lead to ketosis and acidosis. Thus, the overlapping condition known as HHS-DKA may represent a spectrum rather than a distinct disease entity. Although our study did not investigate the temporal sequence of DKA and HHS features, identifying common factors, such as older age, infection, and renal dysfunction, may provide insight into patients at risk for coexisting DKA and HHS. Future research should explore this overlap's underlying mechanisms and progression more thoroughly.

The cornerstone of treatment for DKA and HHS is intravenous fluid and insulin administration [9]. Although rapid fluid loading in pediatric DKA cases can cause cerebral edema [19] and careful treatment is needed, in adult cases, it is rare, and it is recommended to administer isotonic saline (0.9% NaCl) at 500-1000 mL/hour for the first two to four hours. Insulin administration is effective for suppressing hyperglycemia and glucagon secretion, lipolysis, and ketogenesis, and it is recommended to start continuous infusion at 0.1 units/kg/hour [1,9]. In this study, blood glucose level at the start of CII was higher in HHS and HHS-DKA than in DKA (Figure 2). The three groups had no differences in insulin dosage or infusion rate. Still, initial re-examination and final blood glucose levels were significantly higher in HHS-DKA than in the other groups. These results suggest that the response to CII may be lower in the HHS-DKA group than in the DKA or HHS alone group. If HHS-DKA is identified during emergency response, it effectively monitors blood glucose and fluid volume more carefully and is aware of potential complications.

In treatment for DKA or HHS, it is recommended that the insulin infusion rate be adjusted so that blood glucose levels decrease at a rate of 50 mg/dL/hour [6], and potassium monitoring is required every two hours when insulin is infused [20]. In this study, we used machine learning to create a clinical model for predicting hypokalemia after the start of CII. One of this study's strengths is that entering values always clinically evaluated at the beginning of CII makes it easy to predict the risk of hypokalemia after treatment initiation. There have been no previous reports on the development of predictive indicators for hypokalemia in hyperglycemic emergencies, and we hope that this study will serve as a valuable tool for non-specialists with limited experience in the initial management of such cases. Furthermore, early identification of patients at high risk for hypokalemia through our clinical model can enable personalized treatment planning. For example, potassium-containing infusion fluids can be selected earlier in the treatment course. This is particularly important in settings where frequent electrolyte monitoring, such as during nighttime hours or in resource-limited facilities, is not feasible. This approach may improve patient safety and reduce the risk of delayed correction of hypokalemia during continuous insulin infusion therapy. However, a limitation of the clinical model developed in this study is the small number of cases and the lack of external validation. To apply the model in clinical practice, further adjustments are necessary to improve its accuracy using an external validation cohort.

There are several limitations in this study. First, this is a single-center, retrospective observational study of Japanese patients who visited a higher-level medical institution with multiple full-time diabetologists, and the results may differ depending on the region or race. Second, machine learning models cannot extrapolate beyond the training data distribution, and the model in this study was not externally validated. Additionally, instances of normoglycemic ketoacidosis were not included, which may limit the comprehensiveness of the analysis, given the increasing use of SGLT2 inhibitors. Third, the relatively small number of patients in the DKA group (n = 14) may reduce the statistical power of subgroup comparisons. Effect sizes and confidence intervals were not systematically reported, which may limit the interpretation of clinical significance. Non-significant trends, such as gastrointestinal symptoms, should be interpreted with caution. No multivariate analysis was performed to adjust for potential confounders in the observed associations. Fourth, although five-fold cross-validation was applied to reduce overfitting, the limited dataset size still raises concerns about the model’s generalizability. The manuscript did not include key performance metrics such as area under the ROC curve (AUC), sensitivity, specificity, or calibration plots, limiting the ability to assess model performance objectively. Finally, the predictive model remains in an early developmental stage and was internally validated only. External validation using independent datasets will be essential to assess its clinical utility and robustness in broader populations.

We clarified the clinical features of the HHS-DKA combination by comparing them to those with DKA or HHS alone. To our knowledge, few studies have reported on the treatment response and complications of HHS-DKA. In addition, we developed an indicator to predict hypokalemia after the start of CII at our hospital. Suppose the indicator developed in this study is applied clinically. In that case, it will increase the likelihood of receiving appropriate medical care as early as possible after diagnosis, primarily when a doctor rather than a diabetologist performs the initial treatment. Therefore, it is likely that the data in this study are critical and valuable from a clinical point of view. This study has both conceptual and clinical significance; however, the results should be interpreted as preliminary and hypothesis-generating rather than definitive. As this research lays essential groundwork for developing AI-based tools in the acute management of diabetes, future studies with external validation using multicenter cohorts are warranted to assess generalizability and clinical applicability.

## Conclusions

In conclusion, this study's findings highlight the unique and severe clinical nature of HHS-DKA and demonstrate that predictive models for post-treatment complications, such as hypokalemia, may support safer and more efficient care during hyperglycemic emergencies, even when managed by non-specialists in real-world settings.
